# Evaluation of Changes in Effluent Quality from Industrial Complexes on the Korean Nationwide Scale Using a Self-Organizing Map

**DOI:** 10.3390/ijerph9041182

**Published:** 2012-04-11

**Authors:** Mi-Jung Bae, Jun-Su Kim, Young-Seuk Park

**Affiliations:** Department of Biology, Kyung Hee University, Dongdaemun, Seoul 130-701, Korea; Email: mjbae@khu.ac.kr (M.-J.B.); no01knight@nate.com (J.-S.K.)

**Keywords:** water quality, industrial waste, heavy metals, spatial and temporal variation, seasonal Mann-Kendall test, Self-Organizing Map

## Abstract

One of the major issues related to the environment in the 21st century is sustainable development. The innovative economic growth policy has supported relatively successful economic development, but poor environmental conservation efforts, have consequently resulted in serious water quality pollution issues. Hence, assessments of water quality and health are fundamental processes towards conserving and restoring aquatic ecosystems. In this study, we characterized spatial and temporal changes in water quality (specifically physico-chemical variables plus priority and non-priority pollutants) of discharges from industrial complexes on a national scale in Korea. The data were provided by the Water Quality Monitoring Program operated by the Ministry of Environment, Korea and were measured from 1989 to 2008 on a monthly basis at 61 effluent monitoring sites located at industrial complexes. Analysis of monthly and annual changes in water quality, using the seasonal Mann-Kendall test, indicated an improvement in water quality, which was inferred from a continuous increase in dissolved oxygen and decrease in other water quality factors. A Self-Organizing Map, which is an unsupervised artificial neural network, also indicated an improvement of effluent water quality, by showing spatial and temporal differences in the effluent water quality as well as in the occurrence of priority pollutants. Finally, our results suggested that continued long-term monitoring is necessary to establish plans and policies for wastewater management and health assessment.

## 1. Introduction

Water is one of the most essential natural resources, in both quantity and quality, for living organisms, including human beings. Furthermore, water resources are important for the operation of machines and facilities in industries. This natural resource is also important in our economic and social development process [1]. However, rapid industrial development, economic growth, and population growth have intensified the requirements for a vast number of materials and products, leading to an increase in the number of factories in various places across the World. Consequently, available water resources have been reduced, while the environmental pollution of open water systems has increased. However, recently, social concerns and the requirement for environmental conservation have increased across the World, with rising economic standards, which has led to the establishment of wastewater treatment facilities near the industrial complexes for the efficient control of wastewater. 

In addition, to improve environmental conditions and ecological integrity, many countries have developed and modified various laws and regulations. For instance, in the United States, the Federal Water Pollution Act, or Clean Water Act (CWA), was established in 1972 to control the nation's surface waters. In 1972, the CWA set the goal of “restoring and maintaining the chemical, physical, and biological integrity of nation's waters” in the United States [2]. Initially, the CWA aimed to eliminate pollutant discharge into navigable waters by 1985 and to obtain “fishable and swimmable” waters by 1983. Furthermore, in 1977, the National Pollutant Discharge Elimination System was established to limit the discharge of various water pollutants according to effluent standard. The final major amendment to the law was the Water Quality Act (WQA) in 1987 [3]. The WQA emphasized the importance of not violating the environmental standard for water quality in each state (e.g., the Total Maximum Daily Load [TMDL]). In addition, the Storm Water Permit was established to prevent the discharge of large quantities of pollutants from industries or city sewage during rainfall. A similar goal of attaining good ecological status was established by the Water Framework Directive (WFD) in the European Union (EU) from 1973 onwards [4]. On the basis of Kallis and Butler’s study [5], the EU water policy is divided into three periods. In the first period (1973–1986), two water-related directives were implemented: water use directives (e.g., standards for drinking [6], bathing [7], and fish and shellfish harvesting [8,9]) and water pollutant directives (e.g., the standard for the discharge of particular pollutants). The second period (1978–1992), which was marked by the Maastricht Treaty, broadly focused on pollution from urban wastewater [10] and nitrate pollution from agricultural run-off. In addition, certain industrial sectors were regulated by the Urban Wastewater Treatment Directive. Finally, the third period (1993–2000) was represented by the development of the WFD. The goal of the WFD is to achieve a “good surface water status” and “good groundwater status,” as well as to prevent the deterioration in the quality of the already “good” water by 2015 [11]. 

In Korea, the industrial structure has been reorganized across time, with a diverse range of pollutants being emitted from industrial complexes. Consequently, there have been various attempts to improve the efficient management of industrial wastes, such as strengthening emission standards and the types of emissions under regulation. For example, the regulation of effluent quality standard for industrial waste management was first established under the Environmental Pollution Prevention Act in 1963 [12]. Then, the constitution was revised under various directives, such as the Environmental Protection Law (1978), the Law for the Prevention of Water Quality (1991), and the Water Quality and Ecosystem Conservation Act (2007). 

Furthermore, to comprehensively survey the water quality status of streams and lakes at a national scale, water quality has been monitored through the Water Quality Monitoring Networks (WQMN) by the Ministry of Environment in Korea since 1978 (http://water.nier.go.kr). Even though the WQMN has recently expanded to assess Total Pollutant Load Management and Integrated Watershed Management [13], existing research on water quality using the WQMN database is primarily focused on specific regions or specific types of industry [12,14]. 

Understanding how effluent quality of industrial complexes has changed over extended timeframes is fundamental for the effective management of water quality and aquatic ecosystems. Although many studies on the changes of water quality in open water systems exist, research about the changing trends in effluent quality of industrial areas at a national scale are lacking. Hence, the current study aims to: (1) evaluate trends in effluent quality emitted to open water systems from industrial complexes and (2) characterize spatial and temporal differences in wastewater quality on a national scale. We believe that our results will provide the necessary baseline information to set standards for ecological health, water quality conservation, and wastewater management of industrial complexes in Korea.

## 2. Materials and Methods

### 2.1. Water Quality Data

**Figure 1:**
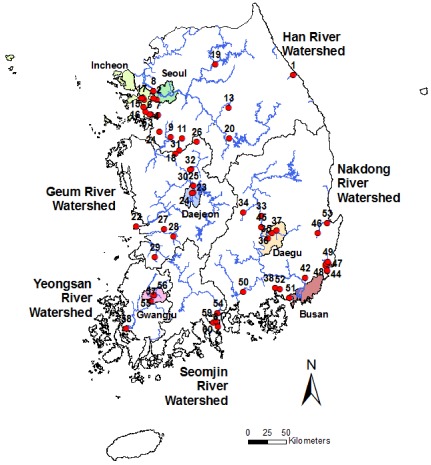
Location of 61 monitoring sites for effluents of the industrial complexes in Korea. Numbers 1–61 represent the monitoring sites listed in Table 1.

Effluent quality data of industrial complexes were obtained from the WQMN database operated by the Ministry of Environment, Korea (http://water.nier.go.kr). From the database, we selected the dataset of 61 monitoring sites based on the survey duration and the data continuity (e.g., monitoring sites including at least 5-year sampling data; Figure 1, Table 1). In the selected dataset, maximum survey periods were for 20 years from 1989 to 2008 (21 of 61 sampling sites). Among 33 water quality factors in the database, we selected 20 factors based on the survey period and measuring frequency: eight physico-chemical factors, including pH, dissolved oxygen (DO), biological oxygen demand (BOD), chemical oxygen demand (COD), total suspended solid (TSS), total nitrogen (TN), total phosphate (TP), and total coliform (TC); nine priority pollutants, including cadmium (Cd), cyan (Cy), lead (Pb), chromium (VI) (Cr^6+^), arsenic (As), mercury (Hg), copper (Cu), zinc (Zn), and phenol; and three non-priority pollutants, including manganese (Mn), iron (Fe), and *n*-hexane (Table 2). All water quality factors were measured based on the standard analytical method [15]. Water samples were typically collected monthly or bimonthly. pH and DO were measured in situ using the appropriate sensors (e.g., pH: 632-pH meter, and DO: TOA DO-149). Other variables were analyzed in the laboratory. Water samples must be preserved and analyzed within the recommended time limit in order to avoid deterioration. BOD measures the amount of oxygen consumed by microorganisms during 5-days incubation in the dark condition at 20 °C. COD was determined from oxidized organic matter in sulfuric acid medium by K_2_Cr_2_O_7_. TSS was determined by the weight difference between before and after filtration through a glass fiber filter, drying at 105–110 °C for two hours. TN and TP were measured by absorptiometry analysis. TC were analyzed by the total coliform fermentation technique or total coliform membrane filter procedure. Cd, Pb, Cr^6+^, As, Hg, Cu and Zn were determined by atomic absorption spectroscopy. Cy and phenol were analyzed based on absorptiometric analysis. Details about the sample measuring protocol are presented in the Korean Ministry of Environment publication [15].

### 2.2. Modeling Changes in Water Quality

A seasonal Mann-Kendall’s test (SMK) [16] was used to evaluate the trend in water quality variables. SMK is regularly used for water quality studies (e.g., [17,18,19,20]), and is a non-parametric and robust procedure that calculates the Mann-Kendall statistic for each user-defined season. Hence, no comparisons are made across season boundaries. In this study, each month represented a “season,” and was evaluated separately; for example, January data are compared only with January, February only with February, *etc*. The R-package *Kendall* was used for SMK, and is available at http://cran.r-project.org.

**Table 1:** Survey periods at the 61 monitoring sites.

Watershed	Site number	Industrial complex	Survey period (year.month)	Watershed	Site number	Industrial complex	Survey period (year.month)	
Han River	1	Gangneung	1992.03–2008.12	Nakdong River	33	Gumi	1992.03-2008.12	
	2	Seoul Digital	1992.03–2008.12		34	Gimcheon	1994.01–2008.12	
	3	Banwol1	1989.02–2008.12		35	Dalseong	1990.01–2008.12	
	4	Banwol2	1989.02–2008.12		36	Daegu 3^rd^	1989.01–2008.12	
	5	Banwol3	1989.02–2008.12		37	Daegu Geomdan	1990.01–2008.12	
	6	Banwol4	1992.03–2008.12		38	Masan	1989.01–2008.12	
	7	Banwol Plating	1994.01–2008.12		39	Petrochemical1	1989.02–2008.12	
	8	Seonam Wastewater treatment	1992.03–2008.12		40	Petrochemical2	1990.01–2008.12	
	9	Songtan	1994.01–2008.12		41	Petrochemical3	1998.02–2008.12	
	10	Sihaw	1995.01–2008.12		42	Yangsan	1989.01–2008.12	
	11	Anseong	1992.03–2008.12		43	Onsan1	1989.01–2008.12	
	12	Yeongdeungpo Mechanical	1992.03–2008.12		44	Onsan2	1989.02–2008.12	
	13	Wonju	1989.01–2008.12		45	Waewan	2001.02–2008.12	
	14	Incheon 5, 6	1992.03–2008.12		46	Yonggang	1994.01–2008.12	
	15	Incheon	2002.01–2008.12		47	Ulsan1	1989.01–2008.12	
	16	Incheon Namdong	1992.03–2008.12		48	Ulsan2	1998.02–2008.12	
	17	Incheon local	1992.03–2008.12		49	Ulsan3	1998.02–2008.12	
	18	Cheonheung	1995.01–2008.12		50	Jinju	1989.01–2008.12	
	19	Chuncheon	1989.01–2008.12		51	Jinhae Macheon	1994.01–2008.12	
	20	Chungju	1998.02–2008.12		52	Changwon	1989.01–2008.12	
	21	Hyangnam Pharmaceutical	1992.03–2008.12		53	Pohang	1989.01–2008.12	
Geum River	22	Gunsan	2003.01–2008.12	Yeongsan River/Seumjin River	54	Gwangyang	1990.01–2008.12	
	23	Daejeon	1989.01–2008.12		55	Gwangju1	2003.01–2008.12	
	24	Daejeon	1989.01–2008.12		56	Gwangju2	2003.01–2008.12	
	25	Daejeon	1994.01–2008.12		57	Gwangju3	2003.01–2008.12	
	26	Daepung	1998.02–2008.12		58	Daebul	2002.01–2008.12	
	27	Iri	1989.01–2008.12		59	Yeocheon1	1999.01–2008.12	
	28	Jeonju	1989.01–2008.12		60	Yeocheon2	1989.01–2008.12	
	29	Jeongeup	1990.01–2008.12		61	Ocheon	1990.01–2008.12	
	30	Cheongju1	1989.07–2008.12					
	31	Cheongju2	1992.01–2008.12					
	32	Hyungdo	1995.01–2008.12					

**Table 2:** Characteristics of water quality variables in the study areas.

Variables	Survey period	Samples/year	Mean	SE *	Valid N **
*Physico-chemical variables*					
DO (mg/L)	1989–2008	24	5.11	0.07	959
BOD (mg/L)	1989–2008	24	40.77	1.81	959
COD (mg/L)	1989–2008	24	36.98	1.18	959
TSS (mg/L)	1989–2008	24	32.59	1.12	959
TC *** × 10^5^ (mg/L)	1989–2008	12	47.0	16.3	959
TN (mg/L)	1994–2008	12	21.72	0.75	776
TP (mg/L)	1994–2008	12	1.51	0.07	777
*Priority pollutants*					
Cd (μg/L)	1989–2008	12	3.42	0.36	959
Cy (μg/L)	1989–2008	12	28.56	5.68	959
Pb (μg/L)	1989–2008	12	17.44	2.23	959
Cr^6+^ (μg/L)	1989–2008	12	1.51	0.31	959
As (μg/L)	1989–2008	12	1.77	0.19	959
Hg (μg/L)	1989–2008	12	0.03	0.01	959
Cu (μg/L)	1989–2008	12	170.21	35.06	959
Zn (μg/L)	1989–2008	12	352.42	43.28	941
Phenol (μg/L)	1989–2008	12	50.73	7.43	941
*Non-priority pollutants*					
Mn (μg/L)	1989–2008	12	294.5	15.19	941
Fe (μg/L)	1989–2008	12	683.17	33.12	941
*n*-Hexane (μg/L)	1989–2008	12	1,325.45	60.01	941

* SE: standard error; ** Valid N: valid number of monitoring samples; *** TC: total coliform.

To characterize water quality factors in terms of spatial and temporal differences, we applied a Self-Organizing Map (SOM) [21,22]. SOM is an unsupervised artificial neural network learning algorithm that approximates the probability density function of the input data [22]. SOM consists of input and output layers that are connected with computational weights (connection intensities). The array of input neurons (computational units) operates as a flow-through layer for the input vectors, whereas the output layer consists of a two-dimensional network of neurons arranged in a hexagonal lattice.

In the SOM learning process, the input data (*i.e*., nine priority pollutants in this study) were initially subjected to the network. The number of output neurons was set to 150 (= 10 × 15) in a 2D hexagonal lattice, which was based on experience and a preliminary study. Subsequently, the weights of the network were trained for a given dataset. Each node of the output layer computes the summed distance between weight vector and input vector. The output nodes are considered as virtual units that represent typical patterns of the input dataset assigned to their units after the learning process [23]. Among all virtual units, the best matching unit (BMU), which has a minimum distance between weight and input vectors, is the winner. For the BMU and its neighborhood units, the new weight vectors are updated by the SOM learning rule. This results in the network being trained to classify the input vectors by the weight vectors to which they are closest. For the training SOM, we used the functions provided in the SOM toolbox [24] of Matlab for Windows ver. 6.1 [25].

We used two different methods to cluster the trained SOM units into several groups. First, the unified distance matrix algorithm (U-matrix; [26]) was applied. The U-matrix calculates distances between neighboring map units, with these distances being visualized to represent clusters using a grey scale display on the map. A hierarchical cluster analysis using Ward's linkage method based on the Euclidean distance measure [27] was applied to the weights of the SOM output units [23,28]. After defining the clusters in the SOM analysis, Multi-Response Permutation Procedures (MRPP) were conducted to test whether there is a significant difference among clusters by using the PC-ORD for Windows ver. 4.25 [29].

In this study, nine priority pollutants (including Cd, Cy, Pb, Cr^6+^, As, Hg, Cu, Zn, and phenol) were selected as input variables in the SOM analysis, based on measuring pollutant continuity. Priority pollutants that had an effect on bioaccumulation, persistency, and carcinogens (even at low concentrations) comprise a set of chemical pollutants that are regulated by the EPA [30].

The Kruskal-Wallis (K-W) [31] test was conducted to evaluate differences in environmental factors for different clusters defined in SOM, and Dunn’s multiple comparison tests were carried out if significant differences in the K-W test (*p* < 0.05) were detected, using statistical software STATISTICA for Windows ver. 7 [32].

## 3. Results

### 3.1. Changes in Water Quality

**Figure 2:**
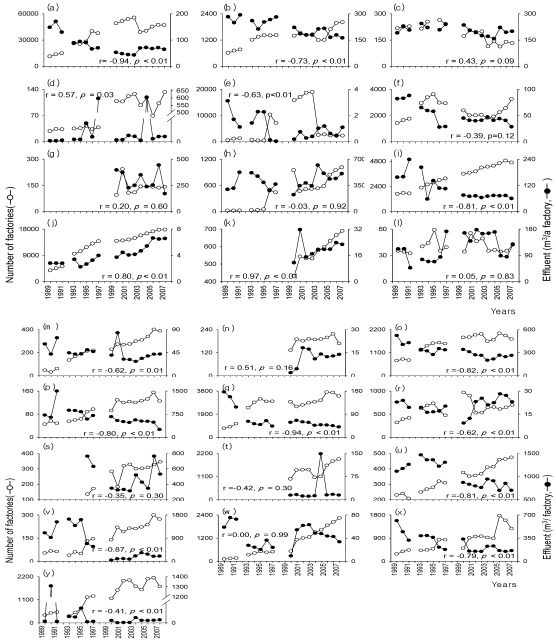
Annual changes both in the number of wastewater discharge factories and in the amount of effluent per factory. (**a**) total; (**b**) chemicals and chemical products; (**c**) leather and allied products; (**d**) wastewater treatment, waste discharge, and cleaning-related services; (**e**) printing and related support activities; (**f**) primary metal manufacture; (**g**) water purification plant; (**h**) electric and electronic manufacture; (**i**) food and drink manufactures; (**j**) repair and maintenance services; (**k**) research and development in the physical, engineering, and life sciences; (**l**) seafood product preparation and packaging; (**m**) dry cleaning and laundry services; (**n**) condensation of cleaning facilities of waste gas; (**o**) clothing manufacture; (**p**) refined petroleum products; (**q**) non-metallic mineral products; (**r**) hospital facility; (**s**) water supply and irrigation systems; (**t**) plate work and fabricated structural products; (**u**) tobacco, pulp, paper, and wood products; (**v**) charcoal, petroleum, and uranium mining; (**w**) rubber and plastic products; (**x**) metal production and processing; and (**y**) others.

We considered annual changes in number of factories discharging wastewater and the amount of wastewater effluent per factory. We found that the amount of effluent per factory tended to decrease, despite an increase in the number of the operational factories for most types of industry (Figure 2). The amount of effluent from the charcoal, petroleum, and uranium mining industries (MCPUI) sharply decreased during the 2000s (*i.e*., 1,177.4 m^3^/factory in the 1990s and 164.7 m^3^/factory in the 2000s). The electric and electronic manufacture industries (MEEI) produced high amounts of effluent during the survey periods, with a major increase in the number of factories after 1995 (*i.e*., 495 factories). Despite there being a large number of factories in the repair and maintenance industry (range 3,771–17,752 factories), the amount of effluent per factory was relatively low (range 2.2–6.6 m^3^/factory). However, the amount of effluent per factory continuously increased, despite low effluent levels. In addition, despite a continuous decline in the amount of effluent per factory, the tobacco, pulp, paper, and wood products manufacture industries (MTPPWPI), the refined petroleum products manufacture industry (MRPPI), the clothing manufacture industry (CMI) and the water treatment industry (WTI) represented relatively high levels of annual effluents (MTPPWPI: 697.3–1,465.9, MRPPI: 248.4–1,497.9, CMI: 199.0–520.9, and WTI: 169.9–442.2 m^3^/factory). 

**Figure 3:**
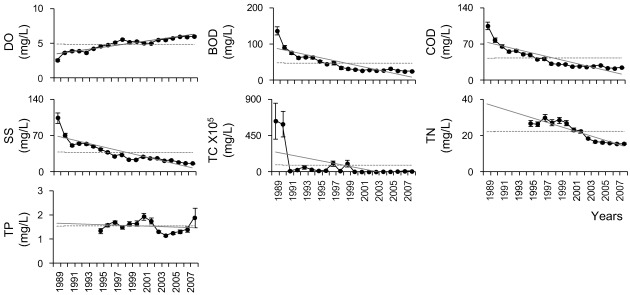
Annual changes in physico-chemical variables from 1989 to 2008. (•: annual mean, Ⅰ: standard error, —: trend line, --: total mean during the survey periods).

**Figure 4:**
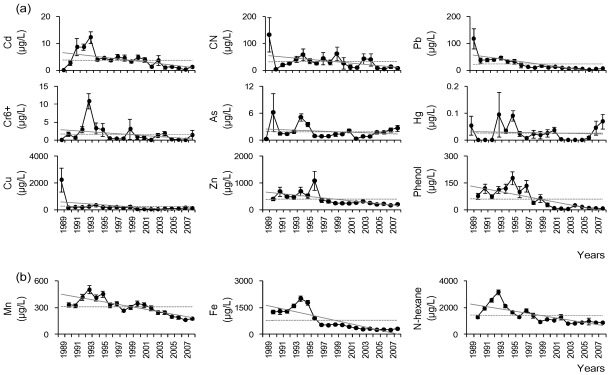
Annual changes in (**a**) priority and (**b**) non-priority pollutants from 1989 to 2008. (•: annual mean, Ⅰ: standard error, —: trend line, --: total mean during the survey periods).

Second, we considered annual changes in water quality variables (*i.e*., physico-chemical variables, as well as priority and non-priority pollutants) from 1989 to 2008. Most physico-chemical variables were generally improved (Figure 3). For example, annual average values of DO tended to increase, while those of BOD, COD, TSS, TC, TN, and TP continuously decreased. Even though the annual average values of priority and non-priority pollutant variables showed high variation compared to physico-chemical variables, there was a general improvement (Figure 4).

**Figure 5:**
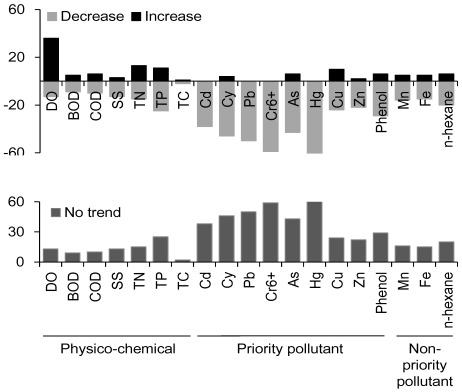
Trends in water quality variables at the 61 monitoring sites of the industrial complexes based on seasonal Mann-Kendall’s test (*p* < 0.05).

SMK test results based on monthly data also showed that the water quality of most effluents improved (Figure 5). For example, upward trends in DO (indicating improving water quality) were found at 36 out of 61 sites, while downward trends were observed for other physico-chemical factors, including BOD (47 sites), COD (45 sites), TSS (45 sites), TN (33 sites), TP (25 sites), and TC (58 sites). 

Out of the nine priority pollutants, decreasing trends were recorded for Cd (23 sites), Cu (27 sites), Zn (37 sites), and phenol (26 sites). In comparison, no significant increasing or decreasing trends were recorded for Cy (46 sites), Pb (50 sites), Cr^6+^ (59 sites), As (43 sites), and Hg (61 sites). All of the non-priority pollutants, such as Mn (40 sites), Fe (41 sites), and *n*-hexane (35 sites) tended to decrease at most of the sites.

### 3.2. Pattern of Water Quality Changes

**Figure 6:**
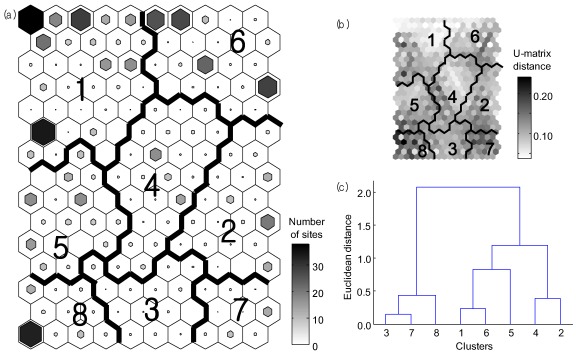
Patterning spatial and temporal changes of effluent quality based on nine priority pollutants at the 61 monitoring sites of the industrial areas in Korea from 1989 to 2008. (**a**) Classification of the trained samples in the SOM with nine priority pollutants; (**b**) U-matrix, and (**c**) Dendrogram of a hierarchical cluster analysis of the SOM units using Ward’s linkage method based on Euclidean distance.

Differences in water quality at 61 monitoring sites across a 20-year period were characterized using nine priority pollutants on the basis of their similarities in SOM (Figure 6). The SOM output units were further classified into eight clusters (1–8) based on the U-matrix, as well as a hierarchical cluster analysis using Ward’s linkage method with Euclidean distance measures. MRPP showed significant differences in the quantity of the nine priority pollutants among eight clusters (A = 0.17, *p* < 0.001). The clusters showed differences in the nine priority pollutants among survey sites and years. In general, the relative ratio of samples measured during 1990s was higher in clusters 7 and 8, while the ratio of samples from the 2000s was higher in cluster 1. In addition, most of the sampling sites, including Daepung and Onsan industrial complexes, in which the effluents of nine priority pollutants were relatively low were located in cluster 1. In comparison, monitoring sites, such as Banwol and Nakdong national and Daegu Dying industrial complexes were in cluster 8, with high effluent levels of the nine priority pollutants. 

Other clusters (2 to 6 clusters) included samples mainly surveyed from the mid 1990s and 2000s. In particular, samples from Dalseuong, Daegu 3rd, and Pohang steel industrial complexes, which were surveyed during this period, were included in cluster 6. 

### 3.3. Differences in Water Quality among SOM Clusters

When considering the differences in water quality factors among the eight clusters, water quality primarily declined from clusters 1 to 8 (Table 3). For example, DO was higher in cluster 1, while other physico-chemical factors (including BOD, COD, TSS, TN, and TP) were higher in cluster 8. In contrast, TC was higher in cluster 6. Among the nine priority pollutants, Cy, Pb, Cr^6+^, As, Hg, Zn, and phenol were higher in clusters 7 and 8, which was similar to the physico-chemical variables. Cd was higher in clusters 2 and 8, while Cu was higher in clusters 3 and 8. All non-priority pollutants showed relatively high values in clusters 7 and 8.

**Figure 7:**
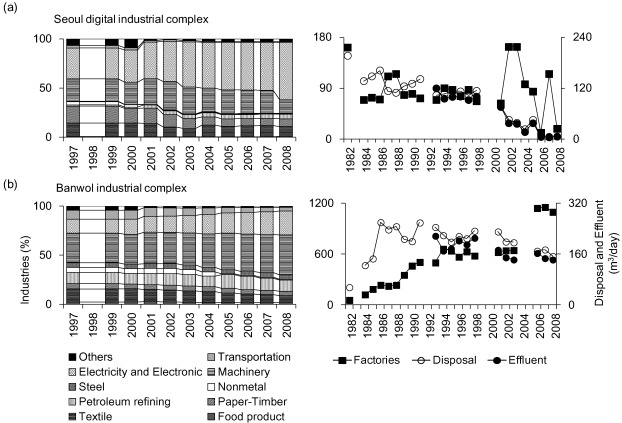
Annual changes in industry types from 1999 to 2008 and temporal changes in wastewater effluent at (**a**) the Seoul Digital industrial complex and (**b**) the Banwol industrial complex.

Annual changes in effluent quality were calculated, and presented in the SOM map (Figure 7). For example, the monitoring sites in the Seoul Digital complex, Banwol Plating industrial complex and the Yeongdeungpo mechanical industrial complex moved from cluster 8 to cluster 1, indicating the recovery process of the nine priority pollutants.

**Table 3:** The mean value and standard error of water quality variables in each cluster defined in SOM. Different alphabet letters indicate significant differences among the clusters based on Dunn’s multiple comparison tests (*p* < 0.05).

Variables	Cluster							
	1	2	3	4	5	6	7	8
Physico-chemical variables								
DO (mg/L)	6.22 (0.12)^a^	5.54 (0.22)^ab^	4.92 (0.39)^bc^	5.18 (0.2)^b^	4.99 (0.18)^b^	4.99 (0.18)^b^	5.13 (0.4)^b^	3.34 (0.28)^c^
BOD (mg/L)	18.41 (2.43)^d^	22.21 (4.61)^cd^	37.08 (8.25)^b^	36.74 (4.29)^bc^	34.74 (3.79)^c^	32.08 (3.69)^c^	43.31 (8.43)^ab^	105.74 (5.78)^a^
COD (mg/L)	20.24 (1.39)^d^	26.15 (2.64)^c^	34.24 (4.74)^b^	28.86 (2.46)^bc^	31.23 (2.17)^b^	30.75 (2.12)^b^	41.91 (4.84)^b^	88.07 (3.31)^a^
TSS (mg/L)	18.42 (1.32)^d^	18.63 (2.5)^cd^	28.77 (4.49)^b^	30.48 (2.33)^b^	28.54 (2.06)^b^	26.39 (2.01)^bc^	28.44 (4.58)^b^	62.06 (3.14)^a^
TC × 10^5^(mg/L)	12.4 (11.2)^c^	2.7 (21.3)^b^	12.5 (38.2)^b^	14.6 (19.8)^b^	10.3 (17.5)^b^	48.8 (17.1)^b^	12.9 (39.0)^ab^	37.8 (26.7)^a^
TN (mg/L)	13.87 (1.1)^c^	22.33 (2.09)^b^	25.03 (3.73)^ab^	22.74 (1.94)^b^	18.48 (1.71)^b^	24.74 (1.67)^b^	39.56 (3.82)^a^	54.49 (2.61)^a^
TP (mg/L)	1.25 (0.11)^b^	1.08 (0.21)^b^	2.1 (0.38)^ab^	1.33 (0.2)^b^	1.87 (0.18)^b^	1.56 (0.17)^b^	1.8 (0.39)^b^	2.65 (0.27)^a^
Priority pollutant variables								
Cd (μg/L)	0.13 (0.43)^f^	11.74 (0.83)^a^	1.59 (1.48)^bc^	0.53 (0.77)^de^	0.41 (0.68)^ef^	2.3 (0.66)^cd^	29.07 (1.51)^a^	0.83 (1.04)^b^
Cy (μg/L)	1.19 (9.22)^e^	1.66 (17.52)^de^	9.98 (31.39)^b^	0.96 (16.3)^d^	15.81 (14.4)^bc^	10.67 (14.04)^cd^	119.1 (32.06)^b^	258.56 (21.97)^a^
Pb (μg/L)	0.44 (2.39)^b^	27.53 (4.53)^a^	21.62 (8.12)^a^	14.58 (4.22)^a^	1.48 (3.73)^b^	0.22 (3.63)^b^	38.49 (8.29)^a^	57.39 (5.68)^a^
Cr^6+^ (μg/L)	0.37 (0.46)^c^	0.01 (0.88)^c^	0.28 (1.57)^abc^	0.35 (0.81)^c^	0.05 (0.72)^c^	0.35 (0.7)^c^	5.22 (1.6)^ab^	8.59 (1.1)^a^
As (μg/L)	2.01 (0.21)^c^	1.21 (0.41)^c^	1.29 (0.73)^bc^	0.36 (0.38)^cd^	0.85 (0.33)^c^	0.07 (0.33)^d^	3.17 (0.75)^ab^	5.47 (0.51)^a^
Hg (μg/L)	0.01 (0.01)^b^	0.06 (0.01)^b^	0.02 (0.02)^b^	0.00 (0.01)^b^	0.02 (0.01)^b^	0.00 (0.01)^b^	0.08 (0.02)^ab^	0.10 (0.02)^a^
Cu (μg/L)	11.8 (28.9)^f^	35.6 (55.0)^cd^	462.7 (98.5)^bc^	45.2 (51.2)^de^	31.3 (45.2)^e^	49.6 (44.1)^c^	176.1 (100.6)^ab^	958.4 (68.9)^a^
Zn (μg/L)	78.9 (75.8)^e^	236.9 (144.1)^bc^	708.4 (258.0)^ab^	172.2 (134.0)^cd^	130.3 (118.4)^d^	239.1 (115.4)^b^	999.8 (263.5)^a^	2,054.9 (180.6)^a^
Phenol (μg/L)	1.5 (12.0)^b^	0.6 (22.8)^b^	29.6 (40.8)^a^	2.4 (21.2)^b^	62.0 (18.7)^a^	0.6 (18.2)^b^	22.7 (41.6)^a^	443.4 (28.5)^a^
Non-priority pollutant variables								
Mn (μg/L)	128.7 (25.1)^c^	418.3 (47.8)^b^	293.4 (85.5)^ab^	200.1 (44.4)^b^	196.2 (39.2)^b^	384.1 (38.3)^b^	1045.4 (87.4)^a^	491.1 (59.9)^a^
Fe (μg/L)	251.0 (40.5)^d^	454.1 (76.9)^b^	544.6 (137.8)^b^	497.3 (71.5)^c^	413.9 (63.2)^c^	568.4 (61.6)^bc^	587.1 (140.7)^ab^	1,903.2 (96.4)^a^
n-hexane (μg/L)	648.4 (92.9)^c^	520.3 (176.6)^b^	1,504.9 (316.2)^b^	1,426.8 (164.2)^b^	1,215.4 (145.1)^b^	1,014.9 (141.4)^b^	1,871.1 (323.0)^b^	3,536.7 (221.3)^a^

**Figure 8:**
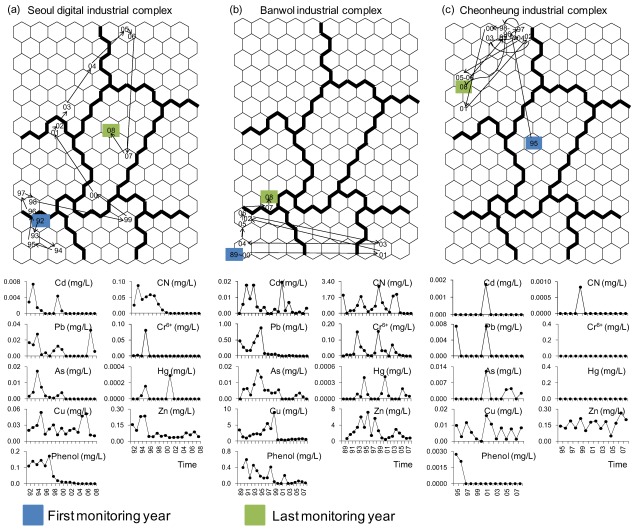
Temporal changes of effluent quality in the SOM ordination and annual changes of nine priority pollutants at (**a**) the Seoul digital industrial complex; (**b**) the Banwol industrial complex; and (**c**) the Cheonheung industrial complex.

In the Seoul Digital industrial complex, which showed an increase in the electronics and electrical industry with a decrease in the annual changes of discharge and effluent (Figure 7a), all samples were located in cluster 8 during the early period (1992–1995), representing relatively high values for priority pollutant variables (Figure 8a). However, these sites moved from cluster 8 to clusters 1 and 4, indicating relatively cleaner states as time progressed. At the Banwol industrial complex, sampling sites were mainly located in cluster 8, and sometimes crossed over to cluster 7 (Figure 8b). The Banwol industrial complex was represented by a high ratio of machinery industry and electric and electronic industries, with a slight decrease in annual discharges and effluent after 1985. Of note, the last two monitoring sites were placed in cluster 5. In contrast, most samples obtained from the Cheonheung industrial complex were located in cluster 1 (Figure 8c).

## 4. Discussion

Since industrial complexes were first built in the 1960s in Korea, industries have mainly focused on economic growth, with limited attention to environmental protection. Industrial complexes that have intensive production activities in a limited space are likely to cause a high occurrence of environmental pollution and issues [33]. However, increasing social concerns and the requirement for the environmental conservation has resulted in the development of stringent environmental standards and legislation being applied to industrial complexes to achieve cleaner production, and a reduction of all types of pollution [33,34,35]. Hence, to effectively manage effluents, it is important to obtain baseline information about the changes in water quality at industrial areas over time. Therefore, in this study, we used SMK and SOM analyses to evaluate changes in water quality at 61 monitoring sites that were located near industrial complexes across Korea (*i.e*., at a national scale) over a 20-year period spanning 1989–2008.

Because key industrial types have changed over time, the amount of effluent according to the industrial types has also changed. In the 1960s, industries were primarily low-skilled, labor intensive, and light, such as the textile and wig industries. This was followed in the 1970s with capital intensive, heavy, and chemical industries, such as steel, machinery, and petrochemical industries. In the 1980s, assembly processing industries, such as consumer-electronics, shipping, and the automotive industry, were dominant. Finally, since the 1990s, the information technology industry, such as the semiconductor industry, computer industry, and telecommunications equipment industry, has been the primary focus of Korean industries. Our results showed that the amount of effluents in MEEI (1990s key industry) was relatively high, with small annual variation. In contrast, the amount of effluent in most other industrial types showed a continuous decline, including the clothing manufacturing industry (1960s key industry), MRPPI (1970s key industry), and metal production and processing industry (1980s key industry). 

Based on annual and monthly changes in water quality, there was a general improvement in effluent quality at most of the 61 monitoring sites. For example, DO gradually increased, while other factors (including BOD, COD, TN, and TP) continuously decreased (Figure 3 and Figure 4). This improvement in water quality might be explained by several legislative efforts in Korea. For instance, water quality standards have strengthened through legal amendments. Since the Environmental Pollution Prevention Act was enacted in 1963, the constitution was amended to the Environment Conservation Act (1978) and Water Quality Conservation Act (1991). However, the water quality control activities were not efficient, because the government mostly focused on industrial and economic development, with limited attention on environmental protection. As economic levels and social development increased, present day social concern and the requirement for the environmental protection strengthened, which led to the development of the Water Quality and Ecosystem Conservation Act (2008). This act has been subject to several amendments that have resulted in the strengthening and refining of the water quality standards, which in turn have contributed to the continuous improvement in water quality. At present, 32 variables are used as parameters to assess water quality. In addition, TMDLs, which differently stipulate the maximum amount of pollutants according to the regions (e.g., watershed, city, or province), have been implemented since August 2004. If the criteria are not met, the local government has the authority to regulate the budget or development work of the industries [36]. The first phase (2004–2010) of TMDL was performed in the Nakdong River basin (from 2004), the Yeongsan River basin (from 2005), and the Seomjin River basin (from 2005). BOD was selected as a criterion for TMDL during the first phase, with the addition of TP during the second phase (2011–2015) [15].

With improvements in the quality of life, recognition and concerns about environmental pollution have changed, along with the policies regarding environmental issues. One of the most representative examples reflecting the power of environmental non-governmental organizations (NGOs) and public response is the Nakdong River phenol emissions. In 1991, citizens in Daegu, which is the third largest city in Korea, identified a foul smell in the tap water [37]. Water supply authorities found that the smell was caused by phenol leakage into the Nakdong River from a large electronics company (more than 30 tons of phenol liquid was released). Hence, the environmental NGOs and citizens organized a phenol investigation team, and boycotted all products from the company at a national scale. Through this incident, public awareness concerning water pollution rapidly increased, and later, an act on special measures for the control of environmental offences was introduced [34].

In addition, as environmental pollution has become a major issue at an international scale, the world community has implemented many comprehensive approaches, including treaties and establishing international organizations to achieve sustainable development. For example, the World Summit on Sustainable Development (WSSD), which took place in Johannesburg, South Africa, in 2002, discussed the issues of Water, Energy, Health, Agriculture, and Biodiversity (WEHAB), along with potential counterstrategies that the international community might take. Such counterstrategies included the increased use of renewable energy, the establishment of a 10-year plan for sustainable production and consumption, and a reduction in the quantity of discharged waste [35]. Furthermore, the World Water Forum is held every three years, and it raises awareness about water-related issues (e.g., water supply and water pollution). At the Rio Earth Summit in 1992, the UN also established World Water Day, which is held on 22 March every year, to focus public attention on critical water issues of our era.

In 2003, the Eco-Industrial Parks (EIP) was established in Korea, which is a national initiative to protect the environment [38]. The objective of the EIP is to minimize the impact of environmental pollution by participating industries and enhance economic outputs. Within the EIP plan, there are three steps to promote 27 EIP for 15 years. Hence, the EIP aims towards developing “green” industry, cleaner production, pollution control, and an increase in energy efficiency, as well as cooperation among companies. Standard industrial complexes only consider raw materials and products, whereas EIP also consider by-product and wastes. Currently, pilot EIP projects are in progress at the Pohang, Yeosu, Ulsan, Banwol, Sihwa, and Cheongju industrial complexes. In 2009, EIP had economic effects of around 340 thousand U.S. dollars, and reduced CO_2_ emissions by about 184,000 tons [35].

The SOM analysis provided a good representation of the observed trends in water quality in this study. Water quality declined from cluster 1, which mainly comprised the 2000s data, to cluster 8, which mainly comprised 1990s data. In addition, the ordination location of each sampling site in the SOM ordination map changed according to the sampling time, reflecting temporal changes in the numbers and types of industrial complexes. For example, the Seoul Digital industrial complex, which moved from cluster 8 to cluster 1 as time progressed, is one of the representative industrial complexes that reflects Korean industrial type changes. During the 1960s, the Seoul Digital industrial complex was mainly focused on export industries (e.g., the textile industry), whereas it has recently been increasingly focused on high-tech industry, information-knowledge based industry, and major company and venture businesses [39]. The venture center was initially constructed by the Korea Industrial Complex Corporation in 2000, with the Seoul Digital industrial complex rapidly shifting into an advanced urban industrial complex. In comparison, at Banwol industrial complex, which was mainly ordinated in cluster 8 for most of the survey period, most priority pollutants tended to decrease along with other physico-chemical variables that also represent an improvement in water quality, despite these variables being relatively higher compared to other industrial complexes. Based on 2008 data, the amount of wastewater discharge (168,845 mg/L, 6.8%) and effluent (156,931 mg/L, 7.5%), as well as the amount of organic loading discharge and effluent, was the highest at this site compared to all other industrial complexes [40]. Even though the ratio of the machinery industry is relatively high, based on Korean standard industrial classification, all types of manufacturing industries are heavily aggregated with small to medium-sized factories, causing difficulty in the efficient management of wastewater. Hence, intensive management, as well as controlling the occurrence of pollution sources, is necessary at the Banwol industrial complex. 

## 5. Conclusions

We have evaluated changes in the spatial and temporal trends of water quality in Korea from 1989 to 2008 using WQMN datasets collected by the Ministry of Environment. When considering annual changes in water quality and SMK, physico-chemical factors, as well as priority and non-priority pollutants, indicated an improvement at monitoring sites for effluents of the industrial complexes. Furthermore, the SOM results also indicated an improvement in water quality, as well as decrease in the amount of effluent from priority pollutants produced by industrial complexes. However, even though water quality showed an improvement, continued long-term monitoring is necessary to establish plans and policies for wastewater management and health assessment in the future.
